# Thromboelastometry and two activated clotting tests in detecting residual heparin after protamine in cardiac surgical patients: A prospective cohort study

**DOI:** 10.1097/EJA.0000000000002122

**Published:** 2025-02-10

**Authors:** Janne Moilanen, Marika Pada, Pasi Ohtonen, Timo Kaakinen, Panu Taskinen, Eeva-Riitta Savolainen, Tiina Erkinaro

**Affiliations:** From the Translational Medicine Research Unit, Medical Research Center Oulu, Oulu University Hospital and University of Oulu, Oulu (JM, MP, PO, TK, PT, TE), Department of Anesthesiology, Vaasa Central Hospital, Wellbeing Services County of Ostrobothnia, Vaasa (MP), Translational Medicine Research Unit, University of Oulu (PO) and Nordlab Oulu Hematology Laboratory, Medical Research Center Oulu, Oulu University Hospital and University of Oulu, Oulu, Finland (E-RS)

## Abstract

**BACKGROUND:**

After cardiac surgery, complete heparin reversal with protamine is essential. Accordingly, there is a need for an accurate and precise point-of-care device to detect possible residual heparin after protamine administration.

**OBJECTIVES:**

To compare two different activated clotting time (ACT) tests and thromboelastometry in detecting postprotamine heparin activity after cardiac surgery.

**DESIGN:**

A single-centre prospective, observational study.

**SETTING:**

University Hospital from September 2021 to February 2023.

**PARTICIPANTS:**

Fifty-five adult, elective cardiac surgical patients.

**INTERVENTIONS:**

The ACT-LR and ACT+ tests of Hemochron Signature Elite device, and the coagulation time (CT) ratio from INTEM and HEPTEM tests of ROTEM Sigma device, were analysed after protamine administration and compared to baseline values.

**MAIN OUTCOME MEASURES:**

Based on postprotamine antifactor Xa (anti-fXa) activity, the patients were divided into heparin (anti-fXa ≥0.2 IU ml^−1^) and no heparin (anti-fXa ≤0.1 IU ml^−1^) groups.

**RESULTS:**

There was a mean bias of 44 [95% confidence interval (CI) 40 to 47] celite seconds between ACT-LR and ACT+ measurements. The absolute changes in ACT-LR, ACT+ and INTEM:HEPTEM CT ratio were variable and did not differ between the groups. The mean ± SD percentage changes between postprotamine and baseline ACT-LR and ACT+ values were 5.9 ± 17.5 and 5.9 ± 16.9% in the no residual heparin group, compared to 1.4 ± 8.4 and 9.9 ± 12.5% in the residual heparin group. Receiver operator characteristic curves for postprotamine INTEM:HEPTEM CT ratio and for percentage changes in ACT-LR and ACT+ to detect an anti-fXa at least 0.2 IU ml^−1^ had areas under the curve of 0.496 (95% CI, 0.329 to 0.663), 0.425 (95% CI, 0.260 to 0.591) and 0.583 (95% CI, 0.417 to 0.749), respectively.

**CONCLUSION:**

Both the ACT-LR and ACT+ tests of Hemochron Signature Elite device and the INTEM:HEPTEM CT ratio of ROTEM Sigma device have poor ability to detect residual heparin shortly after protamine administration.

**KEY POINTS**

- In cardiac surgical patients, increased anti-fXa activity detected 10 to 15 min after protamine administration is associated with incremental postoperative chest tube drainage, underlining the existing need for a reliable point-of-care test to detect residual heparin activity.
- Unfortunately, the ACT-LR and ACT+ tests of Hemochron Signature Elite device, and the INTEM:HEPTEM CT ratio of ROTEM Sigma device, all have poor ability to detect circulating heparin shortly after protamine administration.
- However, the three point-of-care tests are not equivalent, as the sensitivity of ACT+ is better than that of ACT-LR and superior to that of INTEM:HEPTEM CT ratio.

## Introduction

Successful implementation of cardiopulmonary bypass (CPB) necessitates both systemic anticoagulation and effective anticoagulant reversal. Unfractionated heparin for anticoagulation and its specific antagonist protamine sulphate are most often used to accomplish this goal. An adequate dose of protamine after termination of CPB neutralises heparin-induced anticoagulation and reduces the risk of postoperative bleeding, insofar as it is associated with residual heparin activity or heparin rebound. However, excess protamine may contribute to bleeding because protamine itself has anticoagulant properties in the absence of heparin.^1^ Unfortunately, the optimal protamine-to-heparin dosing ratio in cardiac surgery has not been established.^2^

Point-of-care measurements of activated clotting time (ACT) are commonly used in cardiac surgical operating rooms to guide both heparin and protamine administration. It has been suggested in the literature that an increase of at least 10% from baseline to postprotamine ACT values might indicate incomplete heparin reversal.^3^ However, the ACT is not specific to the effects of heparin and correlates only moderately with heparin concentration.^4^ The correlation is further impaired in the presence of common peri-operative factors encountered during cardiac surgery, such as haemodilution, hypothermia, coagulation factor deficiencies, platelet dysfunction, protamine excess, and other medications that impair coagulation and platelet function.^5,6^ Consequently, the capability of ACT to detect residual heparin after protamine reversal has been questioned.^7–9^ Some authors have suggested that viscoelastic tests, such as thromboelastography (TEG) or rotational thromboelastometry (ROTEM), might be more sensitive than ACT in detecting low heparin concentrations,^7,10–12^ but the results are conflicting.^13^

This study was aimed at comparing the performance of three different point-of-care tests available at our institution in detecting a residual heparin effect after protamine administration, as indicated by antifactor Xa activity (anti-fXa) at least 0.2 IU ml^−1^. We studied the Hemochron Signature Elite device using two different ACT test cuvettes, the ACT-LR and the ACT+, and the ROTEM Sigma device. We hypothesised that all three tests are equally sensitive in identifying residual heparin activity after cardiac surgery.

## Materials and methods

This single-centre prospective observational study was conducted in a University Hospital, between September 2021 and February 2023. Ethical approval for this study (EETTMK: 117/2021§) was provided by the Ethical Committee of Oulu University Hospital, Finland on 16 September 2021. Fifty-five adult patients undergoing elective cardiac surgery were included in the study. The exclusion criteria were allergies to heparin or protamine, heparin-induced thrombocytopenia, pre-operative administration of low-molecular-weight heparin, or refusal of the patient to take part. Written informed consent was obtained from all participants.

Pre-operative aspirin was continued to the day of surgery, whereas other antithrombotic agents were stopped at least 5 days before operation. Direct oral anticoagulants and warfarin were discontinued 2 to 3 days pre-operatively. During a pre-operative laboratory visit on the day before surgery, full blood count was performed and prothrombin time (INR, international normalised ratio) and activated partial thromboplastin time (aPTT) were measured.

Our anaesthetic protocol for cardiac surgery has been described in detail previously.^14^ In short, after premedication with diazepam and morphine, general anaesthesia was induced with propofol and remifentanil and maintained by sevoflurane in a mixture of oxygen and air, supplemented with remifentanil and, in some cases, low-dose propofol. A single dose of rocuronium was administered to achieve neuromuscular blockade for endotracheal intubation. A balanced crystalloid solution was used as a first-line agent for intravascular volume replacement, whereas albumin and blood products were combined if deemed necessary by the attending cardiac anaesthesiologist. In patients undergoing CPB, either tranexamic acid or aprotinin was infused for antifibrinolytic effect. Haemodynamic management of the patients also followed our local clinical practice.

During surgery, the patients were anticoagulated using heparin with an initial bolus of 3 mg kg^−1^. ACT+ was measured every 30 min during heparinisation and additional boluses were administered if the target value of more than 480 ‘celite seconds’ for CBP or more than 400 s for off pump surgery was not reached. At the end of the operation, the protamine dose was chosen based on the clinical judgement of the attending cardiac anaesthesiologist, as the study protocol did not include a fixed protamine-to-heparin dosing ratio.

### Coagulation tests

Point-of-care ACT tests were performed using the Hemochron Signature Elite analyser (Instrumentation Laboratory, Bedford, Massachusetts, USA), which automatically aspirates whole blood microsamples into prewarmed test cassettes primed with activators. The instrument recognises clot formation optically when the flow in the cartridge capillaries decreases below a predetermined rate. Paired measurements of ACT were taken using the celite-activated ACT-LR test and the ACT+ cuvette primed with kaolin, silica, and phospholipid. Both tests report a celite equivalent ACT value known as ‘celite seconds’. According to the package inserts, the ACT-LR is designed to measure heparin concentrations of 0 to 2.5 IU ml^−1^, while the ACT+ is calibrated for concentrations of 1 to 6 IU ml^−1^.

Automated thromboelastometry (ROTEM Sigma, Pentapharm GmbH, Munich, Germany) utilising 3.5 ml Vacutainer 3.2% citrated whole blood tubes was performed with a cassette measuring FIBTEM, EXTEM, INTEM and HEPTEM tests. The main ROTEM variables to identify residual heparin are the intrinsically activated INTEM coagulation time (CT) taken in comparison to its heparinase containing equivalent HEPTEM CT. An INTEM : HEPTEM CT ratio of more than 1.1 was considered indicative of residual heparin activity.^15^ In addition to these tests, blood samples were drawn into 2.7 ml Vacutainer 3.2% citrated tubes for the laboratory-based automated chromogenic INNOVANCE anti-fXa assay (Siemens Healthcare GmbH, Germany) to measure anti-fXa, or the antithrombin-catalysed inhibition of factor Xa in plasma. No additional antithrombin is utilised in this assay. In our laboratory, the INNOVANCE assay is calibrated to measure in 0.1 IU ml^−1^ increments anti-fXa activities ranging from 0.1 to 1.5 IU ml^−1^. The cut-off value of anti-fXa ≥ 0.2 IU ml^−1^ was chosen, as only a value of 0.1 IU ml^−1^ or less represents negligible anticoagulation.^16^

### Blood sampling

All blood samples were drawn from an arterial line after discharging 10 ml of aspirated blood. Simultaneous measurements of ACT-LR, ACT+, anti-fXa and ROTEM were taken for baseline and postprotamine values after induction of anaesthesia (but before heparin administration), and 10 to 15 min after protamine administration. In addition, full blood count and INR were analysed after protamine reversal.

After surgery, the patients were taken into the ICU where they were treated according to local clinical practice.^17^ Chest tube drainage was measured hourly.

### Sample size calculation

To achieve an adequate sample size, we collected preliminary data from 40 patients. From this data, we performed the sample size calculation for an equivalence study with the assumption that postprotamine ACT-LR and ACT+ values are comparable. In the preliminary data, the mean postprotamine ACT-LR was 156 celite seconds, and the standard deviation was 17. Assuming the noninferiority margin 10, alpha 0.05 and beta 0.20 (power 0.80), a sample size of at least 50 patients was generated.

### Statistical analysis

The data were analysed using SPSS for Windows (IBM Corp., IBM Statistics for Windows, version 25.0, Armonk, New York, USA). The patients were divided into two groups based on postprotamine anti-fXa value 0.1 IU ml^−1^ or less (no residual heparin group) or at least 0.2 IU ml^−1^ (residual heparin group). Univariate intergroup comparisons for parametric, nonparametric and categorical data were made using Student's *t* test, the Mann–Whitney *U* test or Pearson's *χ*^2^ test, respectively. For baseline and postprotamine coagulation test results, analysis of variance for repeated measurements was used to evaluate whether there were changes in measurements over time within a group, fixed differences between the groups or differences in changes over time between the groups. The association between postprotamine anti-fXa value and cumulative chest tube drainage at 4 h after ICU admission was modelled using linear regression analysis. Receiver operating characteristics (ROC) curves were constructed for postprotamine INTEM:HEPTEM CT ratio and for percentage change between baseline and postprotamine ACT-LR and ACT+ values to detect an anti-fXa at least 0.2 IU ml^−1^. For each ROC curve, the area under the curve (AUC) with 95% CI was calculated. The sensitivities and specificities (with 95% CI) of at least 10% increase from baseline to postprotamine ACT-LR and ACT+ values to detect an anti-fXa at least 0.2 IU ml^−1^ were calculated. The Bland–Altman plot was used to assess the mean bias, with limits of agreement (LoA) and 95% CI, between ACT-LR and ACT+ measurements, whereas proportional bias was evaluated by calculating the regression coefficient.

We present normally distributed data as mean ± SD, nonparametric data as median [IQR], and categorical data as number (%). Two-tailed *P* values are given.

## Results

Altogether, we recruited 55 patients to participate in the study. Due to missing postprotamine anti-fXa measurements, four patients had to be excluded, leaving 51 patients for the final analyses. The patient characteristics are presented in Table 1. There were 21 patients in the no residual heparin group and 30 patients in the residual heparin group. The patients in the no residual heparin group were older 71 [63 to 75] years vs. 62 [54 to 68] years (*P* = 0.002) compared to those in the residual heparin group, but there were no other statistically significant differences in pre-operative characteristics between the two groups (Table 1).

**Table 1:** Patient characteristics

Group	No residual heparin (*n* = 21)	Residual heparin (*n* = 30)	*P*
Gender, male	17 (81)	25 (83)	0.83
Age [years]	71 [63 to 75]	62 [54 to 68]	0.002
BMI (kg m^−1^)	27 [24 to 30]	27 [25 to 30]	0.80
ASA classification			0.68
2	0 (0)	1 (3)	
3	13 (62)	19 (63)	
4	8 (38)	10 (33)	
Aspirin in use^a^	13 (62)	12 (40)	0.083
Type of surgery			0.32
OPCAB	3 (14)	3 (10)	
CABG	8 (38)	6 (20)	
Single valve	5 (24)	7 (23)	
Combined procedure	3 (14)	8 (27)	
Ascending aorta	2 (10)	6 (20)	

Values are *n* (%) or median [IQR]. BMI, body mass index; CABG, coronary artery bypass with grafts; OPCAB, off-pump coronary artery bypass.

a Other antithrombotic medication, direct oral anticoagulants and warfarin were discontinued before surgery according to guidelines.

Intra-operative variables are shown in Table 2. The duration of the operation and CPB did not differ significantly between the groups. The patients in the no residual heparin and residual heparin groups received comparable doses of heparin, 406 [353 to 459] mg vs. 406 [359 to 453] mg (*P* > 0.90), and protamine, 200 [200 to 250] mg vs. 200 [200 to 250] mg (*P* = 0.48). The use of antithrombin III concentrate and antifibrinolytic medications was not significantly different between the groups. The amount of surgical bleeding and the need for blood product transfusions were also comparable in the two groups. However, the patients in the no residual heparin group received more crystalloids, 4917 [4082 to 5752] vs. 3144 [2803 to 3485] ml (*P* < 0.001), and salvaged blood, 370 [240 to 995] vs. 244 [0 to 419] ml (*P* = 0.04), than those in the residual heparin group. In addition, albumin was infused to 10 patients in the no residual heparin compared to two patients in the residual heparin *(P* *<* 0.001).

**Table 2:** Intra-operative management

Group	No residual heparin (*n* = 21)	Residual heparin (*n* = 30)	*P*
Operation time (h : min)	6 : 43 [5 : 57 to 7 : 45]	6 : 02 [5 : 27 to 6 : 45]	0.065
CPB time (h : min)	1 : 56 [1 : 28 to 3 : 04]	2 : 03 [1 : 34 to 2 : 28]	0.55
Antifibrinolytic medication [*n* (%)]			0.60
Tranexamic acid	16 (76)	26 (87)	
Aprotinin	3 (14)	2 (7)	
Antithrombin III [*n* (%)]	1 (5)	2 (7)	0.69
Crystalloids (ml)	4917 [4082 to 5752]	3144 [2803 to 3485]	<0.001
Albumin [*n* (%)]	10 (48)	2 (7)	<0.001
Salvaged blood (ml)	370 [240 to 995]	244 [0 to 419]	0.039
RBC units [*n* (%)]			0.25
1	2 (10)	1 (3)	
2	1 (5)	1 (3)	
3	2 (10)	0 (0)	
FFP units [*n* (%)]			0.46
2	1 (5)	1 (3)	
4	1 (5)	0 (0)	
Thrombocyte units [*n* (%)]			0.52
1	0 (0)	1 (3)	
2	2 (10)	2 (7)	
4	1 (5)	0 (0)	
Total amount of heparin (IU)	406 [353 to 459]	406 [359 to 453]	>0.90
First dose of protamine (IU)	200 [200 to 250]	200 [200 to 250]	0.48
Additional protamine [*n* (%)]	4 (19)	1 (3)	0.063
Total amount of bleeding in the OR (ml)	1000 [750 to 1600]	800 [600 to 1300]	0.13

Values are *n* (%) or median [IQR]. The volume of one RBC, FFP and thrombocyte unit is 260, 200 and 244 ml, respectively. CPB, cardiopulmonary bypass; FFP, fresh frozen plasma; RBC, red blood cells.

The results of pre-operative and postprotamine coagulation tests are given in Table 3. Table 4 summarises the absolute differences and percentage changes between postprotamine and baseline ACT-LR and ACT+ measurements. At baseline, the haemoglobin level of the patients in the no residual heparin group was lower compared to those in the residual heparin group, 13.9 ± 1.6 vs. 14.9 ± 1.3 g dl^−1^ (*P* = 0.01), while there were no significant differences in other laboratory parameters (Table 3). During the operation, haemoglobin level and thrombocyte count decreased while INR increased, but the changes were comparable between the groups (Table 3). Importantly, the changes in INTEM:HEPTEM CT, ACT-LR and ACT+ from baseline to postprotamine values were variable and did not differ between the no residual heparin and residual heparin groups (Tables 3 and 4). An increase of at least from baseline to postprotamine ACT-LR values had a sensitivity of 17% (95% CI 7 to 34%) and a specificity of 81% (95% CI 60 to 92) in detecting an anti-fXa at least 0.2 IU ml^−1^, while the corresponding values for ACT+ were 54% (95% CI 36 to 71) and 60% (95% CI 39 to 78), respectively. All patients in both the no residual heparin and residual heparin group had postoperative INTEM:HEPTEM CT ratios less than 1.1, indicating a sensitivity of 0%.

**Table 3:** Pre-operative and postprotamine coagulation tests

	Pre-operative values		Postprotamine values		
Group	No residual heparin	Residual heparin	No residual heparin	Residual heparin	*P*
Haemoglobin (g dl^−1^) ^∗∗^^,^^∗∗∗^	13.9 ± 1.6	14.9 ± 1.3	10.4 ± 1.2	11.4 ± 1.4	0.80
Thrombocytes (10^9^ l^−1^)^∗∗∗^	230 ± 56	229 ± 55	137 ± 50	142 ± 36	0.64
INR^2^	1.1 ± 0.1	1.1 ± 0.1	1.5 ± 0.2	1.4 ± 0.1	0.36
aPTT (s)	26 ± 3	26 ± 2	--	--	
Anti-fXa (U ml^−1^) ^∗∗∗^	0.1 ± 0	0.1 ± 0	0.1 ± 0	0.3 ± 0.1	<0.001
INTEM:HEPTEM CT	1.01 ± 0.03	1.02 ± 0.04	1.00 ± 0.03	1.00 ± 0.03	0.63
ACT-LR (celite seconds)	155 ± 11	153 ± 12	164 ± 22	155 ± 10	0.21
ACT+ (celite seconds)	112 ± 23	108 ± 11	115 ± 10	118 ± 9	0.29

Values are mean ± SD. aPTT, activated partial thromboplastin time; INR, international normalised ratio.

∗∗ *P* < 0.01 between groups.

∗∗∗ *P* < 0.001 within groups.

**Table 4:** Absolute differences and percentage changes between postprotamine and baseline ACT-LR and ACT+ measurements

	Absolute difference (celite seconds)			Percentage change (%)		
Group	No residual heparin	Residual heparin	*P*	No residual heparin	Residual heparin	*P*
ACT-LR	8.2 ± 25.1	1.4 ± 12.6	0.21	5.9 ± 17.5	1.4 ± 8.4	0.22
ACT+	3.5 ± 25.7	9.7 ± 14.2	0.29	5.9 ± 16.9	9.9 ± 12.5	0.35

Values are mean ± SD.

**Fig. 1:**
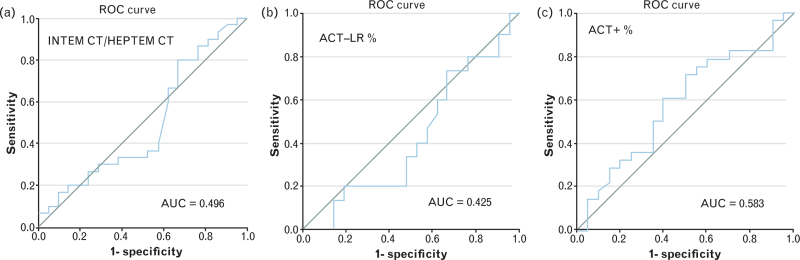
ROC-curves for postprotamine INTEM : HEPTEM CT ratio (a, left panel) and percentage change between baseline and postprotamine values of ACT-LR (b, middle panel) and ACT+ (c, right panel) in detecting residual heparin activity (anti-fXa ≥0.2 IU ml^−1^).

**Fig. 2:**
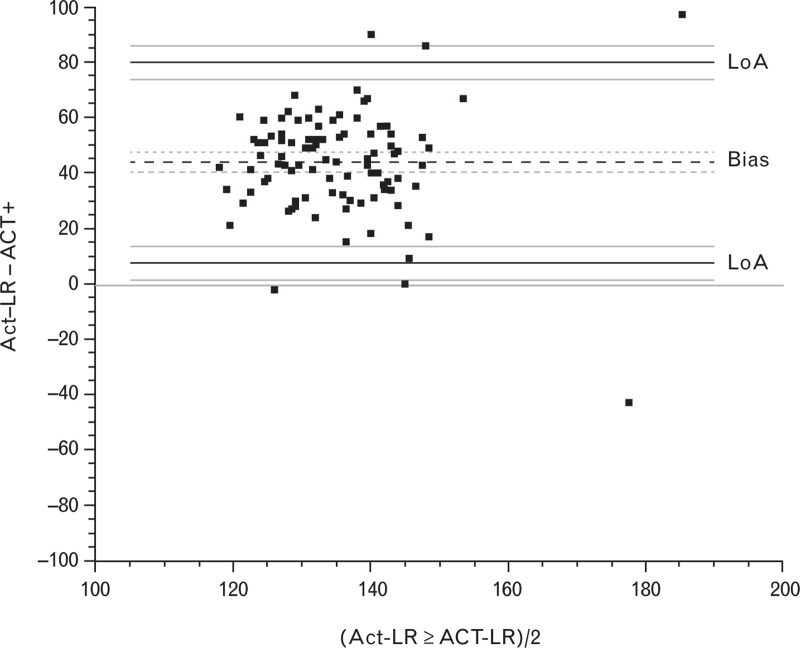
The Bland–Altman plot for baseline and postprotamine ACT-LR and ACT+ measurements.

**Fig. 3:**
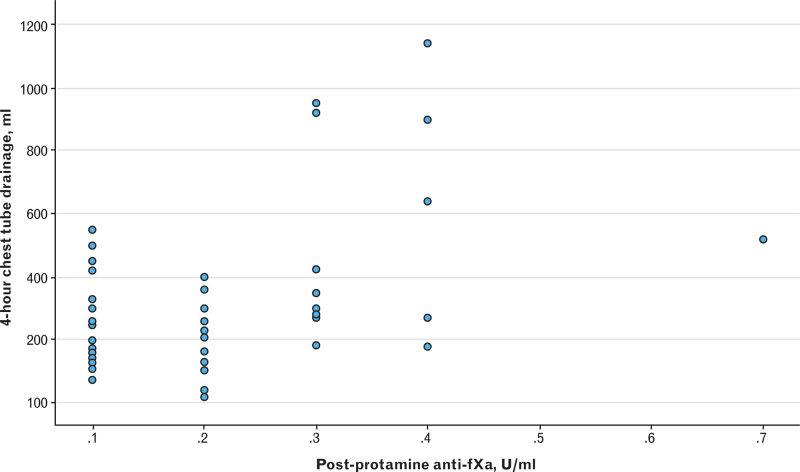
Association of postprotamine anti-fXa to postoperative cumulative 4-h chest tube drainage.

Figure 1 represents the AUC ROC curves for postprotamine INTEM:HEPTEM CT ratio (AUC = 0.496, 95% CI 0.329 to 0.663) and for percentage changes between baseline and postprotamine ACT-LR (AUC = 0.425, 95% CI 0.260 to 0.591) and ACT+ (AUC = 0.583, 95% CI 0.417 to 0.749) to detect an anti-fXa of at least 0.2 IU ml^−1^. The Bland–Altman plot in Fig. 2 demonstrates a mean bias of 44 (95% CI 40 to 47) celite seconds between baseline ACT+ and ACT-LR measurements, which was not proportional to the magnitude of the measurements (regression coefficient 0 celite seconds, 95% CI −84 to 84, *P* > 0.90). Figure 3 shows a scatter plot of the association between postprotamine anti-fXa and cumulative 4-h chest tube drainage. The postprotamine anti-fXa explained 30% (*R*^2^ = 30.3) of the 4-h chest tube drainage. However, the relation was nonlinear with significant second and third power polynomials (Fig. 3).

The lines represent the mean bias and the limits of agreement (LoA) with 95% confidence interval (CI).

Surgical re-exploration was necessary in three patients of the residual heparin group and none in the no residual heparin group (*P* = 0.14). There were no postoperative thromboembolic events nor in-hospital mortality among the study participants.

## Discussion

The main conclusion that can be drawn from our study is that following protamine administration after cardiac surgery, the ability of both Hemochron Signature Elite device and ROTEM Sigma device to detect residual heparin activity is poor. However, in contrast with our hypothesis, the three point-of-care tests are not equivalent as the sensitivity of the ACT+ test appears to be better than that of the ACT-LR test and superior to that of the INTEM:HEPTEM CT ratio. Our results suggest that increased anti-fXa activity detected 10 to 15 min after protamine administration is associated with incremental postoperative chest tube drainage in the ICU. Although postoperative bleeding is known to be multifactorial, our findings underline the existing need for a more reliable point-of-care test to detect residual heparin effect in cardiac surgical patients.

We used anti-fXa rather than aPTT as the indicator of residual heparin activity, because chromogenic anti-fXa assay is an automated laboratory test, which is considered the gold standard to measure actual heparin concentrations.^15,18–20^ It is less influenced than aPTT by confounding factors, such as congenital or acquired coagulation factor deficiencies.^19,21^ Point-of-care testing devices using protamine titration of heparinised blood could also be used to detect residual heparin after protamine reversal.^22^ The HepconHMS device offers a whole blood heparin concentration test. However, its channel resolution is limited to 0.4 IU ml^−1^, and the accuracy suffers from the lack of resolution between cartridges.^12,23^ In addition, anti-fXa has been used as a reference also when validating the HepconHMS device.

We could not find any scientific evidence from the literature to confirm the expert opinion that a difference of at least 10% between baseline and postprotamine ACT measurements might indicate incomplete heparin reversal.^3^ There is some evidence that the accuracy of ACT-LR test in the diagnosis of persisting anticoagulation after vascular surgery is increased when comparing the postoperative measurement to the baseline value.^24^ Yet, this approach is challenging in cardiac surgical patients because ACT is prolonged not only in the presence of heparin but also is affected by several other factors that are common during cardiac surgery,^9^ such as haemodilution, hypothermia, coagulation factor deficiencies, platelet dysfunction, and protamine excess.^5,6,25^ In addition, there is a lot of variation in precision among different ACT monitoring devices.^26,27^ Due to the lack of an accurate, precise, fast, and simple point-of-care test for residual heparin activity, however, various ACT tests are still commonly utilised in cardiac surgical operating rooms to ensure not only sufficient intra-operative heparinisation but also its reversal after surgery.

According to the manufacturer, the celite activated ACT-LR test is designed to measure low heparin concentrations of 0 to 2.5 IU ml^−1^, while the ACT+ cartridge primed with kaolin, silica and phospholipid is optimised to higher heparin concentrations of 1 to 6 IU ml^−1^. A previous study in patients undergoing vascular surgery suggested that ACT-LR measurements are accurate at anti-fXa activities 0.8 IU ml^−1^ or less.^28^ One might, therefore, intuitively expect that the performance of ACT-LR to detect low heparin concentrations also in cardiac surgical patients could be better than that of ACT+. Our results do not support this idea but, instead, challenge the rationale of using the ACT-LR test for postprotamine measurements in this patient group. In patients with residual heparin activity, the mean percentage changes between postprotamine and baseline ACT-LR and ACT+ values were approximately 1 and 10%, respectively. In addition, the sensitivities of at least 10% difference between baseline and postprotamine measurements to detect residual heparin activity were 17 and 54% for ACT-LR and ACT+, respectively. Furthermore, ROC curves for percentage changes between baseline and postprotamine ACT-LR and ACT+ values to detect an anti-fXa at least 0.2 IU ml^−1^ had AUCs of 0.425 and 0.583, respectively. This indicates that ACT-LR performs even worse than random guessing while ACT+ may have at least minimal predictive capability.^29^ The fact that aprotinin prolongs celite-activated ACT measurements^30^ hardly explains the weak performance of the ACT-LR cuvette, as only 5 out of 51 patients received aprotinin. In addition, if aprotinin was used, it was initiated before the blood samples for baseline ACT measurements were drawn, which diminishes the possible effect on the postprotamine vs. baseline ACT-LR difference. Interestingly, a previous study has suggested that there is less variation in celite compared to kaolin-activated measurements at baseline while the difference vanishes during cardiac surgery.^31^ Accordingly, the present results in cardiac surgical patients might in part be explained by the fact that ACT-LR was originally designed for detecting low heparin concentrations in other patient groups with less confounding factors.^28,32^ We speculate that the slightly better ability of the ACT+ test to discriminate residual heparin after cardiac surgery is related to the three distinct coagulation activators in the cuvette, which in the absence of heparin act together to rapidly initiate coagulation despite the presence of confounders, such as the more evident haemodilution in the no heparin group of the present study.

It must be emphasised that the ability of ACT+ to detect residual heparin was also limited, as the changes from baseline to postprotamine values were not statistically significant and did not differ between the no residual heparin and residual heparin groups. This is in line with a previous study in which the ACT+ test was not able to detect incomplete heparin reversal as verified by increased anti-fXa levels.^7^ Other ACT monitoring methods, especially the i-STAT, may show better concordance of repetitive ACT measurements in cardiac surgical patients.^26,33^ However, we are aware of only one publication comparing ACT+ and i-STAT measurements with anti-fXa activity during cardiac surgery.^34^ The authors found that the correlation of both ACT+ and i-STAT values with anti-fXa was acceptable, but the postprotamine values were not separately assessed.^34^ Accordingly, it is not known whether the performance of the i-STAT device, or any other ACT test or device, outweighs that of the ACT+ test in recognising postprotamine heparin activity.

In the present study, we found a mean bias of 44 celite seconds between ACT-LR and ACT+ measurements, which remained constant over the range of measured ACT values. However, the LoA were relatively wide, suggesting limited precision of each test. These results are well in keeping with previous studies comparing various ACT measuring methods and support earlier conclusions that different ACT tests and devices cannot be used interchangeably.^9,26,33–36^

The ability of an older version of thromboelastometry device, ROTEM delta, to detect heparin concentrations ranging from 0.1 to 1.0 IU ml^−1^ has been well documented in an in vitro study by Mittermayr *et al.*,^37^ in which the INTEM : HEPTEM CT ratio increased dose-dependently with increasing heparin concentrations. A study in patients undergoing cardiac surgery suggested that ROTEM delta might be useful in excluding the possibility of residual circulating heparin in patients with prolonged ACT after protamine administration.^10^ Both studies concluded that in protamine excess, INTEM CT and HEPTEM CT are equally prolonged whereas INTEM : HEPTEM CT is not affected.^10^ Another manually operated viscoelastic haemostatic assay, TEG 5000, was shown to allow the diagnosis of postprotamine anti-fXa activity.^7^ Demailly *et al.*^16^ studied the performance of three modern, automated point-of-care devices in cardiac surgical patients and concluded that ROTEM Sigma, TEG 6S and Quantra were equally effective in detecting residual heparin, that is, anti-fXa greater than 0.1 IU ml^−1^. However, anti-fXa was measured only from 15 samples in which the median [IQR] activity was 0 [0 to 0.15] IU ml^−1^.^16^ As there were only few blood samples with elevated heparin concentrations, the results do not allow definitive conclusions on the true performance of ROTEM Sigma in detecting residual heparin activity.

In our study on ROTEM Sigma, we were not able to identify anti-fXa activities at least 0.2 using the INTEM:HEPTEM CT ratio as the ROC curve had an AUC of 0.496. Furthermore, irrespective of the anti-fXa value, none of the patients had a postoperative INTEM : HEPTEM CT ratio greater than 1.1, which was the predetermined limit considered to indicate residual heparin activity.^38^ We were surprised by the finding. As ROTEM Sigma is an automated, cartridge-based system, which overcomes the problem of variability in the manual pipetting technique, it is unlikely that the poor performance was operator-dependent. Quality control and maintenance of our equipment is provided on a regular basis, reducing the risk of technical errors. Protamine excess as a confounding factor is also an unlikely explanation, as the median amount of protamine administered in each group was approximately 50% of the total heparin dose. A previous study with a protamine-to-heparin ratio of roughly 1 : 1 has suggested that there is a transient ‘blind spot’ immediately after protamine administration when ROTEM is not able to detect residual heparinisation,^13^ but this finding has not been confirmed by others. Overall, whether the modern cartridge-based automated viscoelastic tests are as sensitive to heparin as the previous manually operated assays remains to be examined in future studies.

Our study has several limitations. The final study cohort consisted of 51 patients, in which anti-fXa was 0.2 IU ml^−1^ in 16 patients, 0.3 IU ml^−1^ in eight patients, 0.4 IU ml^−1^ in five patients and 0.7 IU ml^−1^ in one patient. It is likely that in a larger sample, the proportion of patients with significantly elevated residual heparin concentration would have been higher, giving more power to the study and allowing stronger conclusions. We used only one Hemochron Signature Elite device for ACT measurements in each patient and cannot exclude a device-related bias in ACT values. However, both ACT-LR and ACT+ tests were performed with the same device. Furthermore, the patients were operated in three different operating rooms, which all have their own devices. Except for routinely measured ACT-LR in the ICU, we did not utilise point-of-care tests in the postoperative assessment of our patients. This limits the generalisability of our results to possible heparin rebound during the postoperative period in the ICU.

## Conclusion

After cardiac surgery, heparin reversal with protamine is of paramount importance. Accordingly, there is a need for an accurate and precise point-of-care device to detect possible residual heparin effect after protamine administration. Our results suggest that the ACT-LR and ACT+ tests of Hemochron Signature Elite device, and the INTEM : HEPTEM CT ratio of ROTEM Sigma device, all have poor ability to detect circulating heparin shortly after protamine administration. Future studies with a larger sample of postprotamine anti-fXa measurements should aim to compare these tests to other ACT and viscoelastic devices with diverse detection methods.
